# A Pharmacoepidemiologic Approach to Evaluate Real-world Effectiveness of Hormonal Contraceptives in the Presence of Drug–drug Interactions

**DOI:** 10.1097/EDE.0000000000001302

**Published:** 2020-11-16

**Authors:** Amir Sarayani, Joshua D. Brown, Amie J. Goodin, Patrick Squires, Phuong Pham, Brian Cicali, Carl Henriksen, Stephan Schmidt, Almut G. Winterstein

**Affiliations:** From the a Department of Pharmaceutical Outcomes and Policy, College of Pharmacy, University of Florida, FL; b Center for Drug Evaluation and Safety, University of Florida, FL; c Department of Pharmaceutics, College of Pharmacy, University of Florida, FL; d Center for Pharmacometrics and Systems Pharmacology, University of Florida, FL.

**Keywords:** Anticonvulsant, Bipolar disorder, Comparative effectiveness, Contraception, CYP3A4, Epilepsy

## Abstract

Accurate estimation of conception is critical in the assessment of the effects of drugs used during pregnancy or to prevent pregnancy. In a novel application, we studied the effectiveness of oral contraceptives (OCs), where misclassification of conception relative to OC exposure may obscure effect estimates.

**Methods:**

We studied OC failure, in a large claims database, among women who used antiepileptic drugs with metabolizing enzyme-inducing properties (carbamazepine or oxcarbazepine), which reduce OC’s effectiveness or enzyme-neutral properties (lamotrigine or levetiracetam), with no expected impact on OC effectiveness. We compared conception rates in women 12–48 years of age concomitantly using OCs and enzyme-inducing drugs with rates in concomitant users of OCs and enzyme-neutral drugs. We measured conception with a validated algorithm that estimates gestational age based on pregnancy endpoints. We estimated relative and attributable risk using generalized estimating equation models after standardized mortality ratio weighting.

**Results:**

We identified 89,777 concomitant use episodes with adjusted contraceptive failure rates of 1.6 (95% confidence interval (CI) = 1.4, 1.8) per 100 person–years among users of enzyme-neutral drugs and 18,964 episodes with a rate of 2.3 (1.9, 2.8) among users of enzyme-inducing drugs. The relative risk of conception for enzyme-inducing group was 1.4 (1.1, 1.8), and the rate difference was 0.7 (0.2, 1.2).

**Conclusions:**

OCs in combination with antiepileptic drugs that interact with metabolic enzymes were associated with increased contraceptive failure rates. Measurement of conception in claims data had adequate accuracy to uncover a strong drug–drug interaction, offering promise for broader application in comparative effectiveness studies on hormonal contraceptives to inform clinical and regulatory decisionmaking.

Observational study designs employing real-world data are commonly used to evaluate the safety and effectiveness of medications during pregnancy.^1,2^ To ensure accurate timing of drug exposure, pregnancy episodes are usually determined via a specific pregnancy endpoint, estimation of gestational age at the time of the endpoint, and imputation of the pregnancy start date.^3–5^ Although the International Classification of Disease (ICD) coding offers detail on gestational age at delivery, the codes are not consistently applied to medical encounter claims, thus leaving some uncertainty about the exact date of conception. Such inaccuracies are more pronounced for preterm deliveries and other adverse pregnancy endpoints such as stillbirths and abortions.^6,7^ Inappropriate timing of conception will in turn result in misclassification of drug exposure, and introduce bias. Misclassification bias will be most pronounced if the exposure window to be studied is close to conception (such as when evaluating effects of first-trimester exposure on the risk for malformation), or exposure pattern varies over time.^8^ It will be even more prominent in scenarios where the pregnancy was not intended or when studying drugs with safety concerns regarding use during pregnancy, and thus exposure is terminated as soon as pregnancy is discovered, leading to only a short period of fetal exposure during pregnancy.

Another research area where accurate timing of conception is critical is in the evaluation of contraceptive effectiveness. Contraceptive failure is operationalized as conception during exposure to the contraceptive agent, and if such failure occurs, the contraceptive use will be discontinued as soon as pregnancy is discovered. Delayed conception estimates may inaccurately conclude that the contraceptive was effective. On the other hand, estimates that time conception too early may conclude that the contraceptive failed even though it may have been discontinued because intentions to prevent pregnancy had changed. Thus, even small errors of conception estimation of only a few weeks, which would be expected for some live births and to a larger extent for nonlive pregnancies, may yield claims data unusable to evaluate the real-world effectiveness of contraceptives. In the previous study, we developed a pregnancy identification algorithm based on several validation studies^6,7^ to evaluate the effectiveness of regulatory requirements designed to prevent maternal exposure to teratogenic medications.^9^ We have recently expanded the algorithm to incorporate diagnosis and procedure codes from the ICD-10. In light of the above-described concerns about conception timing, we aimed to investigate whether pregnancy identification algorithm can accurately identify contraception failure. We chose a clinical scenario where a well-documented drug–drug interaction modifies the failure rate of hormonal contraceptives. In this scenario, possible misclassifications of exposure or outcome could obscure causal inferences when evaluating the drug–drug interaction. However, if shown to be sensitive to detect the drug–drug interaction, this approach would offer opportunities to study a broad range of clinical risk factors for unintended pregnancy using real-world data, including interactions involving hormonal contraceptives, and thus advance pharmacoepidemiologic methods.

## METHODS

### Study Design and Data Source

We conducted a cohort study using the IBM MarketScan Commercial Claims Databases (2005–2017). This database includes data on inpatient and outpatient medical encounters and pharmacy dispensing claims for a large sample of the privately insured population in the United States. The beneficiaries have encrypted identifiers in the database, which allows for longitudinal follow-up. This database is certified as de-identified data, and the present study was approved as exempt by the Institutional Review Board at the University of Florida (IRB approval number: 201801093).

### Clinical Scenario

Carbamazepine and oxcarbazepine are among the first-generation antiepileptic drugs with several approved and off-label indications, including epilepsy and bipolar disorder.^10^ Both drugs are inducers of Cytochrome P450 3A4 (CYP3A4), with well-documented evidence for reduction of estrogen/progestin plasma levels of oral contraceptives (OCs), and ovulation pattern disruption.^11–13^ The enzyme induction effect of carbamazepine and oxcarbazepine is comparable and may result in approximately 50% change in area under the curve (AUC) of OC products. ^12,14^ Therefore, and because both antiepileptic drugs are associated with neural tube defects, clinical guidelines recommend against the use of OCs when using carbamazepine or oxcarbazepine to avoid unintended pregnancy.^15,16^ In contrast, newer antiepileptic drugs, including lamotrigine and levetiracetam, have minimal effect on CYP3A4 and are not expected to reduce OC efficacy.^17,18^ In the present study, we aimed to compare the rate of OC failure in two cohorts of OC users who had concomitant use of either a CYP3A4-inducer (i.e., carbamazepine or oxcarbazepine) or a CYP3A4-neutral (i.e., lamotrigine or levetiracetam) antiepileptic drugs.

### Study Cohorts

We identified female patients of childbearing age (12–48 years old) who had at least one pharmacy claim for a combined OC with low-dose estrogen (<50 µg) or a progestin-only OC, referred to as OCs. We identified pharmacy claims for lamotrigine or levetiracetam to create a cohort of OC users without drug–drug interaction and extracted claims for carbamazepine or oxcarbazepine to create a cohort of OC users with drug–drug interaction. These four antiepileptic drugs were selected based on their profile of CYPA34 activity and potential clinical uses to create comparable study cohorts with regard to baseline clinical characteristics and pregnancy rates. We defined the cohort entry date (i.e., index date) as the first day of concomitant use of the drugs of interest and OCs and defined a look-back period of six months before the index date with continuous insurance enrollment to ascertain drug indications and other covariates. Patients were required to have at least one medical claim for epilepsy, bipolar disorder, or personality disorder (i.e., indications for antiepileptic drugs) during the look-back period. The indications were measured using coding algorithms developed by the Center for Medicare and Medicaid Services, Chronic Conditions Data Warehouse based on the ICD, ninth, and tenth versions, clinical modification (ICD9/10-CM) codes.^19^ We excluded patients if they had medical diagnoses for infertility, ovary dysfunction, or hirsutism in their look back period to rule out off-label indications for OCs. The list of ICD codes is provided in eAppendix 1; http://links.lww.com/EDE/B746.

### Concomitancy Definition

We assumed drug exposure started at the prescription dispensing date and ended on the last day of the pharmacy-entered dispensed days’ supply. We defined “concomitancy” as overlapping exposure periods regardless of the order of drug dispensing for antiepileptic drugs or OCs. ^20^ We excluded concomitancy periods that had exposure to valproate sodium, topiramate, or phenytoin because of potential CYP3A4 activity and teratogenic effects, which may encourage patients to use a second contraceptive method (e.g., barrier methods). We also excluded concomitancy periods where we observed any other hormonal contraceptive agents, including long-acting reversible contraceptives (e.g., intrauterine devices), injectables, or high-dose estrogen OCs. For users of CYP3A4-neutral antiepileptic drugs, we excluded the concomitancy periods with CYP3A4-inducer drugs. Then we created concomitant use episodes for patients in each study group, and a gap of ≥14 days for either of the medications during an episode was allowed. If we observed a gap of more than 14 days concomitant use, the concomitancy episode ended on the last day of concomitant use. Patients were allowed to reenter the cohort if they had subsequent concomitant use episodes after their first observation period and met all inclusion criteria, including the availability of the 6-months look-back period to allow re-evaluation of baseline characteristics.

### Outcome Definition

The study outcome was contraception failure defined as conception during a concomitancy period. Conception was estimated via the pregnancy identification algorithm that uses medical encounters with ICD-9/10-CM, Current Procedural Terminology, and Healthcare Common Procedure Coding System codes to identify specific pregnancy endpoints, including live birth, ectopic pregnancies, stillbirth, terminations, and prenatal screening visits.^6,7,21,22^ Once pregnancy episodes were identified, the algorithm estimated gestational age to calculate the last menstrual period (LMP). The conception date was assumed to be 14 days after the estimated LMP date. We provide more details on the pregnancy identification algorithm in eAppendix 1; http://links.lww.com/EDE/B746.

### Covariates

We measured several demographic and clinical variables at baseline to assess the comparability of study cohorts, including patient age, residence region, insurance plan type, and relationship to the employee covered by the health plan (spouse, employee, children/other). Clinical variables measured during the 6-months look-back period included recent pregnancy history (based on any pregnancy endpoint), use of teratogenic drugs (with or without mandated pregnancy prevention programs), and a variety of clinical conditions that may affect OC efficacy.

### Study Follow-up

All patients were required to have insurance enrollment for a minimum of 90 days after their concomitancy episode ended. This requirement allows the pregnancy identification algorithm to capture pregnancy-related medical encounters, which are then used to date conception. This minimum number of days was defined based on our previous work that showed approximately 90% of live deliveries in our database have prenatal visits within the first 90 days after conception. We followed each patient from the index date of each concomitancy episode until conception, infertility, ovary dysfunction, hirsutism diagnoses, initiation of a teratogenic drug, end of concomitancy, maximum of 3 years’ follow-up, or end of study (December 31, 2017).

### Statistical Analysis

The unit of analysis was a concomitancy episode. We compared baseline demographic and clinical characteristics of each study cohort using a threshold of an absolute standardized difference (ASD) higher than 10% as clinically significant.^23^ To account for confounding, we used a logistic regression model to create an exposure propensity score and selected covariates into the model based on a literature review on potential risk factors for the study outcome.^24^ We used the common support region of the score to create weights to estimate the average treatment effect among the treated, also known as the standardized mortality ratio (SMR) weighting method.^23,25^ In this weighted pseudo-population, the confounding effect of measured covariates is eliminated, and the effect estimates can approximate the causal effect. We used these SMR weights in a generalized estimating equation model with a Poisson distribution and offset of follow-up time to compare contraception failure rates among the study cohort. We used a robust variance estimator to account for the clustering of episodes within the same patient. We conducted all data management and analyses using SAS 9.4 and SAS/STAT 15.1 (Cary, NC).

### Sensitivity Analysis

We performed several sensitivity analyses to evaluate the robustness of the study findings. For the concomitancy definition, we changed the gap allowance to 1 or 7 days instead of 14 days. We also excluded concomitancy periods that overlapped with other moderate or strong inducers or inhibitors of CYP3A4 for more than 14 days (see eAppendix-1; http://links.lww.com/EDE/B746: eTable 1; http://links.lww.com/EDE/B746 and eTable 2; http://links.lww.com/EDE/B746). For the outcome definition, we varied the estimated conception date by ±14 days, limited the analysis to episodes indexed before 2015 (confining coding to ICD-9-CM), limited the outcome definition to only live birth episodes (which allows the most accurate conception date estimation). To evaluate the impact of more homogeneous comparison groups, we restricted the maximum follow-up time to 6 months, limited the analysis to only the first episode of concomitancy for each patient, and restricted drug initiation sequence to patients who initiated OC initiation while on antiepileptic drug treatment.

## RESULTS

In the main analysis, we identified 89,777 concomitancy episodes involving CYP3A4-neutral antiepileptic drug and 18,964 episodes with the CYP3A4 inducers. Cohorts had similar age distributions with a mean age of 26.3 ± 8.5 years for the CYP3A4-neutral and 25.5 ± 9.2 for the CYP3A4-inducing drugs (Table 1). About 70% of women in each cohort had a bipolar disorder diagnosis, and 30% had an epilepsy diagnosis. We observed a high prevalence of anxiety (29% vs. 29%) and teratogenic medication use (30% vs. 28%) in the baseline period. The baseline covariates were balanced between study cohorts except for age, beneficiary status, and paralysis diagnosis (ASD < 15%). SMR weighting successfully balanced all measured characteristics (Table 1). Propensity score distributions, SMR weights, and hazard plots are available in eAppendix 1; http://links.lww.com/EDE/B746.

**TABLE 1.** Demographic and Clinical Characteristics of the Study Cohorts

Covariates	Before SMR Weighting			After SMR Weighting		
	Concomitant OC Plus Enzyme-neutral AED Episodes^a^ (N = 89,777)	Concomitant OC Plus Enzyme-inducing AED Episodes^b^ (N = 18,964)	ASD (%)	Concomitant OC Plus Enzyme-neutral AED Episodes^a^ (N = 18,973)	Concomitant OC Plus Enzyme-inducing AED Episodes^b^ (N = 18,964)	ASD (%)
Age	n (%)	n (%)		n (%)	n (%)	
<20	23,297 (26)	6337 (33)	16	6359 (34)	6337 (33)	0
20–29	36,339 (41)	6748 (36)	10	6741 (36)	6748 (36)	0
30–39	21,618 (24)	3918 (21)	8	3910 (21)	3918 (21)	0
≥40	8523 (10)	1961 (10)	3	1964 (10)	1961 (10)	0
Hypertension	3732 (4)	946 (5)	4	958 (5)	946 (5)	0
Hyperlipidemia	4114 (5)	1011 (5)	3	1021 (5)	1011 (5)	0
Obesity	1277 (1)	357 (2)	4	358 (2)	357 (2)	0
Epilepsy	23,309 (26)	5816 (31)	10	5853 (31)	5816 (31)	0
Bipolar disorder	65,116 (73)	13,081 (69)	8	13,057 (69)	13,081 (69)	0
Schizophrenia	2428 (3)	852 (5)	10	853 (4)	852 (5)	0
Depression	50,723 (57)	10,219 (54)	5	10,215 (54)	10,219 (54)	0
Personality Disorder	5606 (6)	1209 (6)	1	1210 (6)	1209 (6)	0
Anxiety	26,043 (29)	5544 (29)	1	5542 (29)	5544 (29)	0
Substance Use Disorder	4575 (5)	1284 (7)	7	1288 (7)	1284 (7)	0
Recent pregnancy (live birth)	2248 (3)	309 (2)	6	308 (2)	309 (2)	0
Recent pregnancy (termination)	507 (1)	104 (1)	0	103 (1)	104 (1)	0
Teratogenic drug without REMS	24,925 (28)	5588 (30)	4	5620 (30)	5588 (30)	0
Teratogenic drug with REMS	414 (1)	95 (1)	1	96 (0)	95 (1)	0
Charlson Comorbidity Index (CCI)^3^						
≤1	87,223 (97)	18,93 (95)	NA	18,127 (95)	18,093 (95)	NA
1<	2554 (3)	871 (5)	NA	846 (5)	871 (5)	NA
Comorbidities						
Myocardial infarction	31 (0)	< 11	0	<11	< 11	0
Congestive Heart Failure	175 (0)	50 (0)	1	51 (0)	50 (3)	0
Vascular Disorder	187 (0)	51 (0)	1	54 (0)	51 (3)	0
Cerebrovascular Disorder	892 (1)	197 (1)	1	202 (1)	197 (1)	0
Pulmonary Disorders	7211 (8)	1705 (9)	3	1716 (9)	1705 (9)	0
Dementia	86 (0)	30 (0)	2	31 (2)	30 (0)	0
Paralysis	613 (1)	380 (2)	11	388 (2)	380 (2)	0
Diabetes w/o complications	1945 (2)	432 (2)	1	434 (2)	432 (2)	0
Diabetes with complications	175 (0)	40 (0)	0	40 (0)	40 (0)	0
Renal Disorders	263 (0)	64 (0)	1	67 (3)	64 (0)	0
Mild Liver Disorders	765 (1)	192 (1)	2	194 (1)	192 (1)	0
Severe Liver Disorders	22 (0)	<11	0	<11	<11	0
Peptic Ulcer	193 (0)	58 (0)	2	60 (3)	58 (0)	0
Rheumatoid Disorders	585 (1)	156 (1)	2	160 (8)	156 (1)	0
AIDS	22 (0)	<11	0	<11	<11	0
Malignancy	793 (1)	170 (1)	0	173 (9)	170 (1)	0
Metastatic Malignancy	39 (0)	<11	0	<11	<11	0
Beneficiary status						
Employee	32,469 (36)	5702 (30)	13	5698 (30)	5702 (30)	0
Spouse	14,208 (16)	2764 (15)	3	2677 (15)	2764 (15)	0
Child/other	43,100 (48)	10,498 (55)	15	10,509 (55)	10,498 (55)	0
Residence region						
Northeast	16,228 (18)	3045 (17)	5	3047 (16)	3045 (16)	0
Northcentral	19,394 (22)	4363 (23)	4	4370 (23)	4363 (23)	0
South	35,322 (39)	8074 (43)	7	8071 (42)	8074 (43)	0
West	17,594 (20)	3225 (17)	7	3228 (17)	3225 (17)	0
Unknown	1239 (1)	257 (1)	0	257 (1)	257 (1)	0
Health plan type						
COM	1525 (2)	479 (2)	6	482 (3)	479 (3)	0
HMO	12,955 (14)	2749 (14)	0	2733 (14)	2749 (15)	0
PPO	53,996 (60)	11,362 (60)	1	11,373 (60)	11,362 (60)	0
POS	6853 (8)	1594 (8)	0	1597 (8)	1594 (8)	0
CDHP	5887 (6)	1216 (6)	1	1218 (6)	1216 (6)	0
Other	8561 (9)	1564 (8)	5	1570 (8)	1564 (8)	0

ASD, absolute standardized difference; COM, comprehensive; HMO, health maintenance organization; PPO, preferred provider organization; POS, noncapitated point-of-service; CDHP, consumer-driven health plan; Other: includes capitated/partially capitated point-of-service, exclusive provider organization, high deductible health plan; REMS, risk evaluation and mitigation strategy.

^a^Cohort A: concomitant use of oral contraceptives and CYP3A4-neutral drugs (lamotrigine or levetiracetam). After SMR weighting, the size of the pseudo-population in cohort A becomes comparable to cohort B.

^b^Cohort B: concomitant use of oral contraceptives and CYP3A4-inducer drugs (carbamazepine or oxcarbazepine).

**FIGURE 1.**
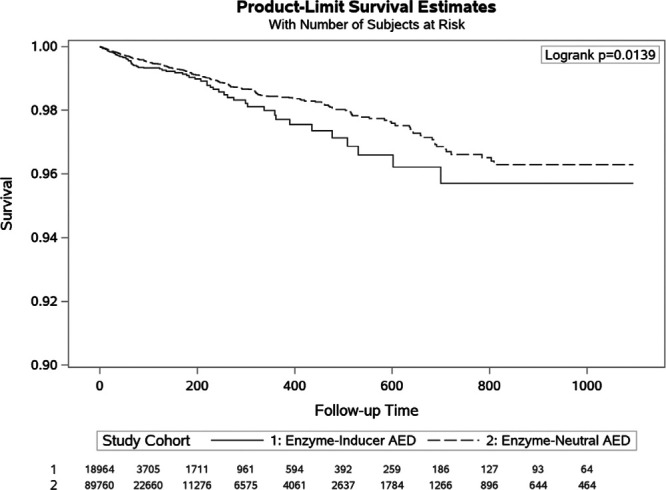
Survival curves for contraception failure during follow-up in each study cohort.

Episodes involving concomitant use of enzyme-neutral antiepileptic drugs had a slightly larger mean follow-up time of 96 days (vs. 79 days among women who used enzyme-inducers). Concomitancy periods ended with 400 conceptions among women who used enzyme-neutral antiepileptic drugs, resulting in a crude contraception failure rate of 1.7 events per 100 person–years. Women with concomitant use of OCs and enzyme-inducing antiepileptic drug had 94 conceptions with a crude contraception failure rate of 2.3 per 100 person–years (Table 2). Figure 1 shows unadjusted survival plots for contraception failure outcome. Approximately two-thirds of all conceptions were identified based on liveborn deliveries (both groups 63%), whereas abortions were the second prevalent pregnancy endpoint (28% in the enzyme-neutral group vs. 27% in the enzyme-inducing group) (Table 3).

**TABLE 2.** Relative and Absolute Risk of Oral Contraceptive Failure in the Presence or Absence of Drug–drug Interaction

Study Cohort	Events	Total Follow-up Time (Person-years)	Incidence Rate (per 100 Person-years)	Relative Risk	Risk Difference
Unadjusted analysis					
Concomitant OC plus enzyme-neutral AED episodes^a^	400	23,647	1.7 (1.5, 1.9)	REF	REF
Concomitant OC plus enzyme-inducing AED episodes^b^	94	4102	2.3 (1.9, 2.8)	1.4 (1.1, 1.7)	0.6 (0.1, 1.1)
Adjusted Analysis					
Concomitant OC plus enzyme-neutral AED episodes^a^	400	23,647	1.6 (1.4, 1.8)	REF	REF
Concomitant OC plus enzyme-inducing AED episodes^b^	94	4102	2.3 (1.9, 2.8)	1.4 (1.1, 1.8)	0.7 (0.2, 1.2)

ASD, absolute standardized difference; OC, oral contraceptive.

^a^Cohort A: concomitant use of oral contraceptives and CYP3A4-neutral drugs (lamotrigine or levetiracetam)—Reference cohort.

^b^Cohort B: concomitant use of oral contraceptives and CYP3A4-inducer drugs (carbamazepine or oxcarbazepine).

**TABLE 3.** Pattern of Pregnancy Episodes that Contributed to Measurement of Contraception Failure

Study Cohort	Full-term n (%)	Preterm n (%)	Post-term n (%)	Ectopic n (%)	Stillbirth n (%)	Spontaneous abortion n (%)	Induced abortion n (%)	Unknown outcome n (%)	Total n
Concomitant OC plus enzyme-neutral AED episodes^a^	233 (58)	16 (4)	3 (1)	4 (1)	3 (1)	65 (16)	46 (12)	30 (8)	400
Concomitant OC plus enzyme-inducing AED episodes^b^	54 (57)	4 (4)	1 (1)	1 (1)	0 (0)	14 (15)	11 (12)	9 (10)	94

ASD, absolute standardized difference; OC, oral contraceptive.

^a^Cohort A: concomitant use of oral contraceptives and CYP3A4-neutral drugs (lamotrigine or levetiracetam)—Reference cohort.

^b^Cohort B: concomitant use of oral contraceptives and CYP3A4-inducer drugs (carbamazepine or oxcarbazepine).

The adjusted contraceptive failure rates were 1.6 (95% CI = 1.4, 1.8) per 100 person–years among users of enzyme-neutral drugs and 2.3 (1.9, 2.8) among users of enzyme-inducing drugs. The marginal models with SMR weights increased the unadjusted relative risk for contraception failure slightly from 1.4 (1.1, 1.7) to 1.4 (1.1, 1.8), comparing women who used enzyme-inducing antiepileptic drugs to those who used enzyme-neutral antiepileptic drug. Concomitant use of enzyme-inducing antiepileptic drug and OCs resulting in an additional 0.7 conceptions (0.2, 1.2) per 100 person–years of concomitant use.

All sensitivity analyses corroborated our findings (Table 4). Analyses with conceivably superior measurement of confounding (analysis number 6, 7, 9 in Table 4) showed as expected slightly larger relative risk estimates with 1.6 (1.2, 2.2) when restricting to the first concomitancy episode per patient, 1.5 (1.1, 2.1) with a fixed sequence of drug initiation, and 1.5 (1.2, 1.9) after eliminating concomitant use of all other potential CYP3A4 inducers/inhibitors.

**TABLE 4.** Sensitivity Analyses on the Definitions of Concomitancy, Outcome Measurement, and Study Design Features

Row	Sensitivity Analysis	Incidence Rate (per 100 Person-year)		Adjusted Rate Ratio	Adjusted Rate Difference
		Concomitant OC Plus Enzyme-neutral AED Episodes^a^	Concomitant OC Plus Enzyme-inducing AED Episodes^b^		
1	Conception date altered by +14 days	1.5 (1.3, 1.7)	1.9 (1.5, 2.4)	1.3 (1.0, 1.6)	0.4 (0.0, 0.9)
2	Conception date altered by −14 days	1.9 (1.7, 2.1)	2.5 (2.1, 3.0)	1.3 (1.1, 1.7)	0.6 (0.1, 1.1)
3	Restriction to episodes with index date before 2015 (ICD-9 era)	1.6 (1.4, 1.8)	2.3 (1.9, 2.9)	1.6 (1.1, 1.8)	0.7 (0.2, 1.3)
4	Conception ascertained based on live birth only	1.0 (0.9, 1.2)	1.4 (1.1, 1.8)	1.4 (1.1, 1.9)	0.4 (0.2, 8.0)
5	Maximum follow-up time restricted to 6 months	1.7 (1.5, 1.9)	2.3 (1.9, 2.9)	1.4 (1.1, 1.8)	0.6 (0.1, 1.2)
6	Episodes restricted to first episode per patient	1.6 (1.4, 1.8)	2.5 (1.9, 3.3)	1.6 (1.2, 2.2)	0.9 (0.2, 1.6)
7	Episodes restricted to those where OC initiation follows AED use	1.5 (1.3, 1.8)	2.3 (1.8, 3.1)	1.5 (1.1, 2.1)	0.8 (0.1, 1.5)
8	Permissable gap in concomitancy 7 days	1.6 (1.5, 1.8)	2.2 (1.8, 2.7)	1.4 (1.1, 1.7)	0.6 (0.1, 1.0)
9	Permissable gap in concomitancy 1 day	1.6 (1.5, 1.8)	2.1 (1.7, 2.6)	1.3 (1.0, 1.6)	0.5 (0.0, 0.9)
10	Exclusion of all follow-up time with other CYP3A4 inducers or inhibitors	1.6 (1.4, 1.8)	2.4 (1.9, 2.9)	1.5 (1.2, 1.9)	0.7 (0.2, 1.3)

ASD, absolute standardized difference; ICD, International Classification of Disease; OC, oral contraceptive.

^a^Cohort A: concomitant use of oral contraceptives and CYP3A4-neutral drugs (lamotrigine or levetiracetam)—Reference cohort.

^b^Cohort B: concomitant use of oral contraceptives and CYP3A4-inducer drugs (carbamazepine or oxcarbazepine).

## DISCUSSION

Our study found that women who concomitantly used OCs and CYP3A4-inducing antiepileptic drugs were 40% more likely to experience contraceptive failure compared to CYP3A4-neutral antiepileptic users. Our findings suggest that conception estimation was sufficiently accurate to identify this well-documented strong drug–drug interaction, and thus, our approach may be useful to generate real-world evidence on mechanisms of contraceptive failure, including the examination of interactions.

Because respective drug approval requirements are largely confined to pharmacokinetic studies, limited information exists on the clinical significance of drug–drug interaction involving OCs. A meta-analysis published in 2010 identified only pharmacokinetic studies that examined the potential for contraceptive failure among patients who use antiepileptic drugs.^26^ We identified one small cohort study published in 1979 that followed 41 epilepsy patients with concomitant use of antiepileptic drugs and OCs and reported ~2.9 failures per 100 person–years, which is slightly higher than in our cohorts.^27^ In our study, we observed a failure rate of 1.6 or 2.3 per 100-person years, depending on the type of antiepileptic drugs in terms of enzyme-inducing properties. The observed magnitude of the drug–drug interaction impact on failure rates in our study is biologically plausible based on evidence from pharmacokinetic evaluations, which recommend higher OC doses when used concomitantly with carbamazepine (e.g., 80–100 mcg of ethinyl estradiol that far exceeds the observed doses of 20–35 mcg observed in our data). ^11,12^ A clinical trial on healthy volunteers showed a 46.1% reduction in the AUC for levonorgestrel, and 44.5% for ethinyl estradiol when the OC was administrated with carbamazepine (600 mg). The study also reported more ovulations (5/10 cycles vs. 1/10 cycles) among carbamazepine users versus OC use alone.^12^ The diminishing effects of CYP3A4 inducers, including carbamazepine and oxcarbazepine, on the efficacy of OC is also emphasized in the guidance document for labeling of combined hormonal contraceptives.^28^

Because clinical trials on contraceptive failure are typically infeasible, observational studies, especially prospective cohorts, have a pivotal role.^29–31^ Broader availability of real-world data and advancements in pharmacoepidemiologic methods can facilitate comparative effectiveness studies and help to translate mechanistic findings into clinically significant outcomes. To the best of our knowledge, the present study is the first attempt to investigate contraceptive failure in claims data. To evaluate the performance of this approach, we designed our study based on a clinical scenario of decreased OC efficacy in the presence of a known drug–drug interaction with a well-studied potent enzyme inducer. We successfully replicated mechanistic findings regarding a significant interaction between OC use and the perpetrator drug (a CYP3A4-inducer), resulting in contraceptive failure. Therefore, we envision that this approach could serve as a novel platform to evaluate the comparative effectiveness of hormonal contraceptives, considering different routes of administration and differences in pharmacokinetic profiles, among diverse patient populations with comorbidities or other concomitant medications. However, researchers should be vigilant about potential misclassification biases in exposure (contraceptive use) or outcome (conception) measurements and their consequences on the ability to make causal inferences, especially, for quantifying the magnitude of risk, and the possibility of inadequate sensitivity of our approach to detect weaker drug–drug interaction effects.

We operationalized exposure in our claims data by using “days of supply” recorded on the pharmacy claims. Although this measurement approach is more reliable than patient self-report, it may not be fully reflective of the actual medication consumption.^32^ For instance, women might discontinue OC treatment before their supply is exhausted to plan for pregnancy. This decision would possibly result in misclassification of exposure and overestimation of the contraception failure rate. The use of active comparator groups that exhibit similar demographics and comorbidities, such as demonstrated in our cohort, may mitigate some of these concerns, but even nondifferential exposure misclassification could bias relative risk estimates either away or toward the null hypothesis (eAppendix 2; http://links.lww.com/EDE/B747).

Regarding outcome measurement, we relied on an algorithm to infer pregnancy episodes from medical encounters with the healthcare system that use validated coding to specify pregnancy endpoints, but without an actual recording of LMP. Based on previous literature, estimation of conception will have varying degrees of accuracy for each type of pregnancy endpoint.^6,22^ In our pregnancy identification algorithm, we assigned gestational age for live-birth episodes based on an algorithm of ICD codes indicating gestational age or preterm status, and the overall agreement against birth certificates is reported to be >93% in the Medicaid database.^22^ For ectopic pregnancy, induced/spontaneous abortion, and stillbirth, we used fixed values for gestational age (8, 10, and 28 weeks, respectively) similar to previous literature.^6,7^ This approach has shown a moderate agreement both against medical charts (70% for ectopic pregnancy and 67% for spontaneous abortions within four weeks)^7^, and a cohort of in-vitro fertilization patients (77% agreement for ectopic pregnancy, 47% for stillbirth, and 36% for abortions cases).^6^ Validation studies on pregnancy identification algorithms typically report the agreement between the estimated LMP and the gold standard (e.g., the clinical estimate of gestational age on the birth certificate) within a prespecified number of weeks (e.g., 2 weeks) as the margin of error.^4,6,22^ It should be noted; however, that such a margin might need to be varied according to the pregnancy endpoint that was used to estimate LMP. For example, previous studies have shown that the capture of preterm status among deliveries improves with decreasing gestational age.^33^ Thus, considering the relationship between measurement sensitivity and gestational age as well as the pronounced left-skewed distribution of gestational age among preterm infants, error margins of 2–4 weeks used in sensitivity analyses may be appropriate. In contrast, stillbirth with a flat gestational age frequency distribution ranging from 20 to 42 weeks may require broader margins that should be tested.^34^ We should also note that available conception algorithms typically estimate LMP, following long-established conventions in timing gestational age, and thus, conception must be imputed as LMP + 14 days to operationalize contraceptive failure.^35^ In our study, we conducted sensitivity analyses by varying the estimated conception date, limiting the analysis to the ICD-9-CM era with previously validated pregnancy endpoint definitions, and restricting the events to pregnancies with the liveborn outcome. The two latter analyses aimed to increase the specificity of the outcome measure.

Our adjustment for potential confounding was based on a literature review on risk factors for contraceptive failure, but we acknowledge that several predictive factors were not or were only partially measurable in claims data. For example, sexual activity may be an important factor for contraceptive failure but is not available in claims data. However, we believe that our use of an active comparator group successfully balanced for the majority of risk factors, as exemplified by fairly well-balanced comparison groups before SMR weighting. We should also acknowledge that race and socioeconomic status were unmeasured in our dataset and could act as confounding factors. However, the study drugs are available on the market as generic products, yielding channeling for economic reasons unlikely. Finally, we should note that our adjustment for confounding further increased the observed relative risk estimates, thus suggesting that enzyme inducers were slightly more prevalent among patients with fewer risk factors for contraceptive failure. Thus, to explain our findings, any unmeasured covariate would need to be distributed in the opposite direction than the measured risk factors. Researchers could apply probabilistic bias analysis methods to evaluate the impact of exposure, outcome, and confounder misclassifications, simultaneously.^36,37^

In conclusion, women who use OCs in combination with carbamazepine or oxcarbazepine should be aware of increased contraceptive failure rates. Our study showed that measurement of conception in claims data has adequate accuracy to reveal the effect of a known drug–drug interaction with hormonal contraceptives. Our pharmacoepidemiologic approach is promising for comparative effectiveness studies on hormonal contraceptives to generate real-world evidence and inform clinical and regulatory decision-making.

## ACKNOWLEDGMENTS

None.

## Supplementary Material


