# Supplementary material for: A Pharmacoepidemiologic Approach to Evaluate Real-world Effectiveness of Hormonal Contraceptives in the Presence of Drug–drug Interactions
Source: Epidemiology. 2020 Nov 16;32(2):268–76. doi: 10.1097/EDE.0000000000001302 (PMC7850590; doi:10.1097/EDE.0000000000001302)

Appendix 1

eTable 1- List of ICD/CPT codes used for measurements of covariates/conditions

eTable 2- List of drugs with moderate/strong induction effect on CYP3A4

eTable 3 - List of drugs with moderate/strong inhibition effect on CYP3A4

eFigure 1- Description of the Pregnancy Identification Algorithm (PIA)

eFigure 2- Distribution of propensity scores in the study cohorts

eFigure 3- Distribution of SMR weights created based on the propensity scores

eFigure 4- The hazard function plot for the contraception failure in each study cohort

eTable 1- List of ICD/CPT codes used for measurements of covariates/conditions

Covariate/Condition	Code*
Infertility diagnosis/procedure (ICD-9)	Dx:<br>6185x, 627x, 628x, 25631, 2562x, V252x, V8801, V4981, V2651<br>Proc:<br>6629, 6661, 6621, 6622,655, 6551, 6552, 6553, 6554, 656, 6561, 6562, 6563, 6564, 683, 6831, 6839, 684, 6841, 6849, 685, 6851, 6859, 686, 6861, 6869, 687, 6871, 6879,689
Infertility diagnosis/procedure (ICD-10)	Dx:<br>E8940x, E8941x, N993x, N950x, N951x, N952x, N958x, N958x, N959x, N970x, E230x, N971x, N972x, N978x, N978x, N979x, E28310, E28319, Z302x, Z9851x, Z780x, Z90710<br>Proc:<br>0UT90ZZ,0UT94ZZ,0UT20ZZ,0UT27ZZ,0UT28ZZ,0UT2FZZ, 0UT00ZZ,0UT07ZZ,0UT08ZZ,0UT0FZZ,0UT10ZZ,0UT17ZZ, 0UT18ZZ,0UT1FZZ,0UT24ZZ,0UT04ZZ,0UT14ZZ,0UT20ZZ, 0UT70ZZ,0UT00ZZ,0UT10ZZ,0UT50ZZ,0UT60ZZ,0UT24ZZ, 0UT74ZZ,0UT04ZZ,0UT14ZZ,0UT54ZZ,0UT64ZZ,0UL74ZZ, 0UL78ZZ,0U574ZZ,0U578ZZ,0UL74CZ,0UL74DZ,0UL74ZZ, 0UL78DZ,0UL78ZZ,0U550ZZ,0U553ZZ,0U554ZZ,0U557ZZ, 0U558ZZ,0U560ZZ,0U563ZZ,0U564ZZ,0U567ZZ,0U568ZZ, 0U570ZZ,0U573ZZ,0U574ZZ,0U577ZZ,0U578ZZ,0UB50ZZ, 0UB53ZZ,0UB54ZZ,0UB57ZZ,0UB58ZZ,0UB60ZZ,0UB63ZZ, 0UB64ZZ,0UB67ZZ,0UB68ZZ,0UT94ZL,0UT90ZL,0UT94ZZ, 0UT94ZZ,0UTC4ZZ,0UT90ZZ,0UT90ZZ,0UTC0ZZ,0UT9FZL, 0UT9FZZ,0UT9FZZ,0UTC4ZZ,0UT97ZL,0UT97ZZ,0UT97ZZ, 0UT98ZL,0UT98ZZ,0UT98ZZ,0UTC7ZZ,0UTC8ZZ,0UT44ZZ, 0UT94ZZ,0UTC4ZZ,0UT40ZZ,0UT90ZZ,0UTC0ZZ,0UT44ZZ, 0UT9FZZ,0UTC4ZZ,0UT47ZZ,0UT48ZZ,0UT97ZZ,0UT98ZZ, 0UTC7ZZ,0UTC8ZZ
Infertility procedure (CPT)	56308, 58951, 58953, 58954,58150,58700,56307, 58150, 58720, 58940, 58943, 58950, 58951, 58952, 58953, 58954, 58600, 58605, 58615, 58611, 58670, 58671, 58565, 58150, 58152, 58180, 58200, 58210, 58240, 58260, 58262, 58263, 58267, 58270, 58275, 58280, 58285, 58290, 58291, 58292, 58293, 58294
Ovary dysfunction (ICD-9)	256x, 2560x, 2561x, 2562x, 2563x, 25631x, 25639, 2564x, 2568x, 2569x
Ovary dysfunction (ICD-10)	E28x, E280x, E281x, E282x, E283x, E2831x, E28310, E28319, E2839x, E288x, E289x
Hirsutism (ICD-9)	7041x
Hirsutism (ICD-10)	L680x
Hypertension (ICD-9)	36211, 4010, 4011, 4019, 40200, 40201, 40210, 40211, 40290, 40291, 40300, 40301, 40310, 40311, 40390, 40391, 40400,

	40401, 40402, 40403, 40410, 40411, 40412, 40413, 40490, 40491, 40492, 40493, 40501, 40509, 40511, 40519, 40591, 40599, 4372,
Hypertension (ICD-10)	H35031, H35032, H35033, H35039, I10, I110, I119, I120, I129, I130, I1310, I1311, I132, I150, I151, I152, I158, I159, I674, N262
Hyperlipidemia (ICD-9)	2720, 2721, 2722, 2723, 2724
Hyperlipidemia (ICD-10)	E780, E781, E782, E783, E784, E785
Obesity/overweight (ICD-9)	2780,27800,27801,27803, V853, V8530, V8531, V8532, V8533, V8534, V8535, V8536, V8537, V8538, V8539, V854, V8541, V8542, V8543, V8544, V8545, 27802
Obesity/overweight (ICD-10)	E6601, E6609, E661, E662, E668, E669, Z6830, Z6831, Z6832, Z6833, Z6834, Z6835, Z6836, Z6837, Z6838, Z6839, Z6841, Z6842, Z6843, Z6844, Z6845, E663

- The decimal point is removed from the codes.

eTable 2- List of drugs with moderate/strong induction effect on CYP3A4

**Drug Name**	**Source**
Carbamazepine	Both
Dexamethasone	Both
Enzalutamide	Both
Lumacaftor	DrugBank
Midostaurin	DrugBank
Mitotane	DrugBank
Phenobarbital	Both
Phenytoin	Both
Rifampicin	Both
Rifaximin	DrugBank
Avasimibe	DrugBank
Bosentan	DrugBank
Dabrafenib	DrugBank
Echinacea	DrugBank
Etravirine	DrugBank
Modafinil	Both
nevirapine	University of Indiana (Flockhart Table)
oxcarbazepine	University of Indiana (Flockhart Table)
pioglitazone	University of Indiana (Flockhart Table)
rifabutin	University of Indiana (Flockhart Table)
troglitazone	University of Indiana (Flockhart Table)
Betamethasone	University of Indiana (Flockhart Table)
Budesonide	University of Indiana (Flockhart Table)
cortisone (cortisol)	University of Indiana (Flockhart Table)

eTable 3 - List of drugs with moderate/strong inhibition effect on CYP3A4

Drug Name	Source
Atazanavir	DrugBank
Boceprevir	Both
Clarithromycin	Both
Cobicistat	DrugBank
Conivaptan	DrugBank
Curcumin	DrugBank
Darunavir	DrugBank
Delavirdine	DrugBank
Econazole	DrugBank
Ergotamine	DrugBank
Idelalisib	Both
Indinavir	Both
Itraconazole	Both
Ketoconazole	Both
Lopinavir	DrugBank
Nefazodone	Both
Nelfinavir	Both
Nilotinib	DrugBank
Posaconazole	DrugBank
Ribociclib	Both
Ritonavir	Both
Saquinavir	Both
Telaprevir	Both
Telithromycin	Both
Terfenadine	DrugBank
Tipranavir	DrugBank
Voriconazole	Both
Aprepitant	Both
Cyclosporine	DrugBank
Diltiazem	Both
Dronedarone	DrugBank
Erythromycin	Both
Fluconazole	Both
Fluvoxamine	Both
Fusidic acid	DrugBank
Haloperidol	DrugBank
Isoniazid	DrugBank
Lovastatin	DrugBank
Luliconazole	DrugBank
Miconazole	DrugBank
Netupitant	DrugBank
Nicardipine	DrugBank
Primaquine	DrugBank

Sertraline	DrugBank
Tioconazole	DrugBank
Verapamil	Both
grapefruit juice	University of Indiana (Flockhart Table)
cimetidine	University of Indiana (Flockhart Table)
esomeprazole	University of Indiana (Flockhart Table)
lesinurad	University of Indiana (Flockhart Table)
pantoprazole	University of Indiana (Flockhart Table)
amiodarone	University of Indiana (Flockhart Table)
chloramphenicol	University of Indiana (Flockhart Table)
ciprofloxacin	University of Indiana (Flockhart Table)
gestodene	University of Indiana (Flockhart Table)
imatinib	University of Indiana (Flockhart Table)
mibefradil	University of Indiana (Flockhart Table)
mifepristone	University of Indiana (Flockhart Table)
norfloxacin	University of Indiana (Flockhart Table)

Methodology:

These tables were generating by extracting drugs classified as moderate or strong inducer/inhibitor of CYP3A4 within the drugbank.ca[1] database. For robustness, similar information was extracted from the Drug Interactions Flockhart Table ™ (University of Indiana)[2] . After adjusting for duplicates, the Drug Interaction Database (DIDB),[3] developed by the University of Washington, was leveraged to identify literature-reported AUC/Cmax changes of the respective drugs to determine a final list of drugs that can potentially affect CYP3A4.

1. https://www.drugbank.ca/
2. https://drug-interactions.medicine.iu.edu/MainTable.aspx
3. https://sop.washington.edu/department-of-pharmaceutics/research/drug-interaction-database/

eFigure 1- Description of the Pregnancy Identification Algorithm (PIA)

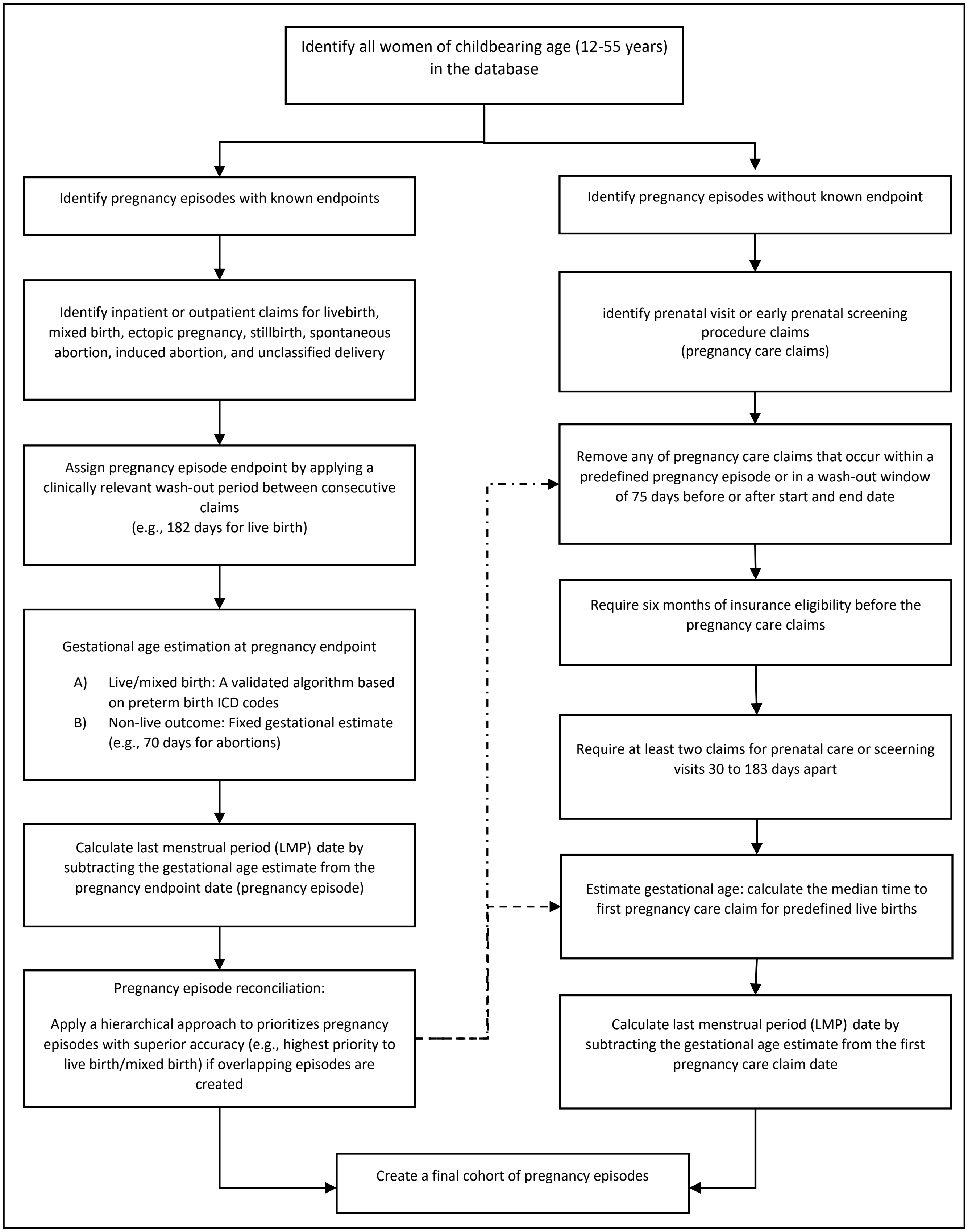

eFigure 2- Distribution of propensity scores in the study cohorts

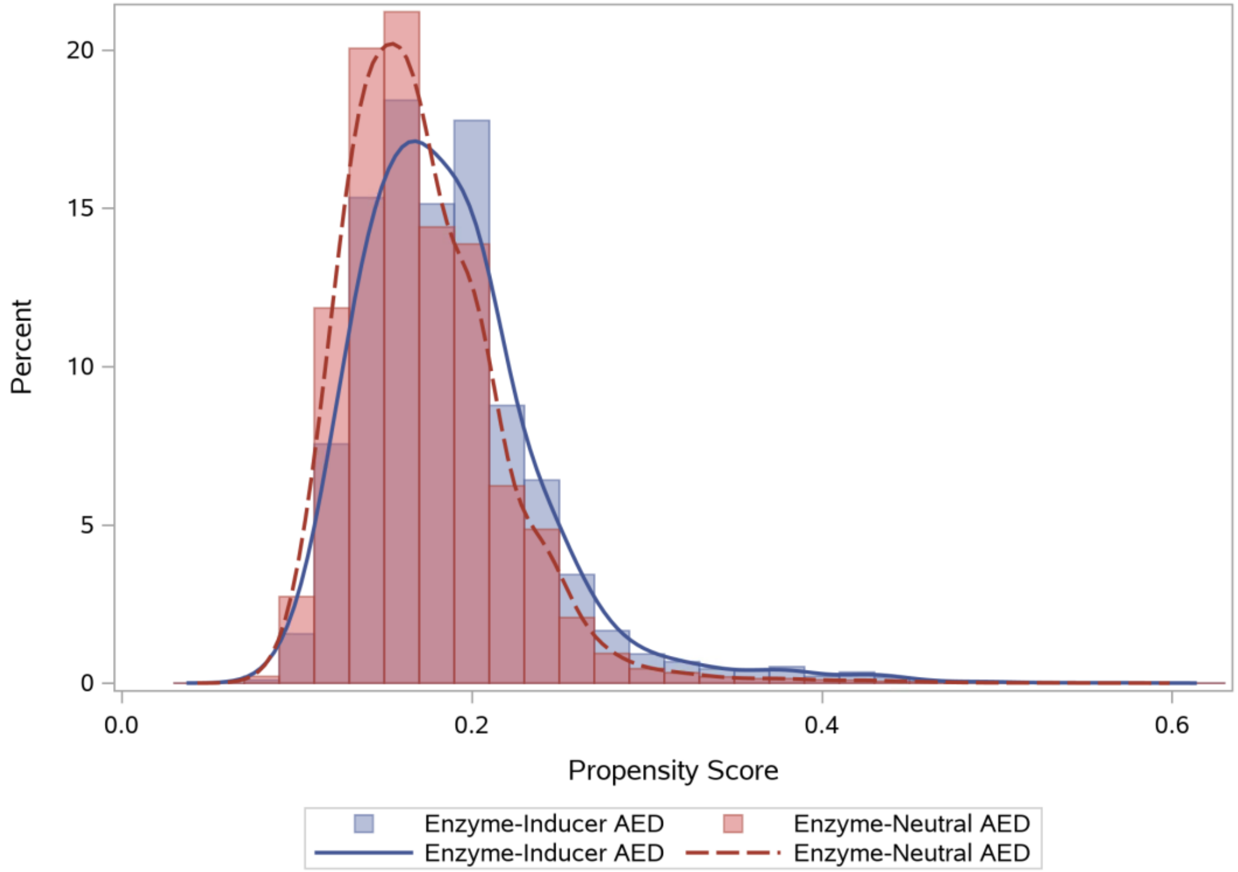

eFigure 3- Distribution of SMR weights created based on the propensity scores

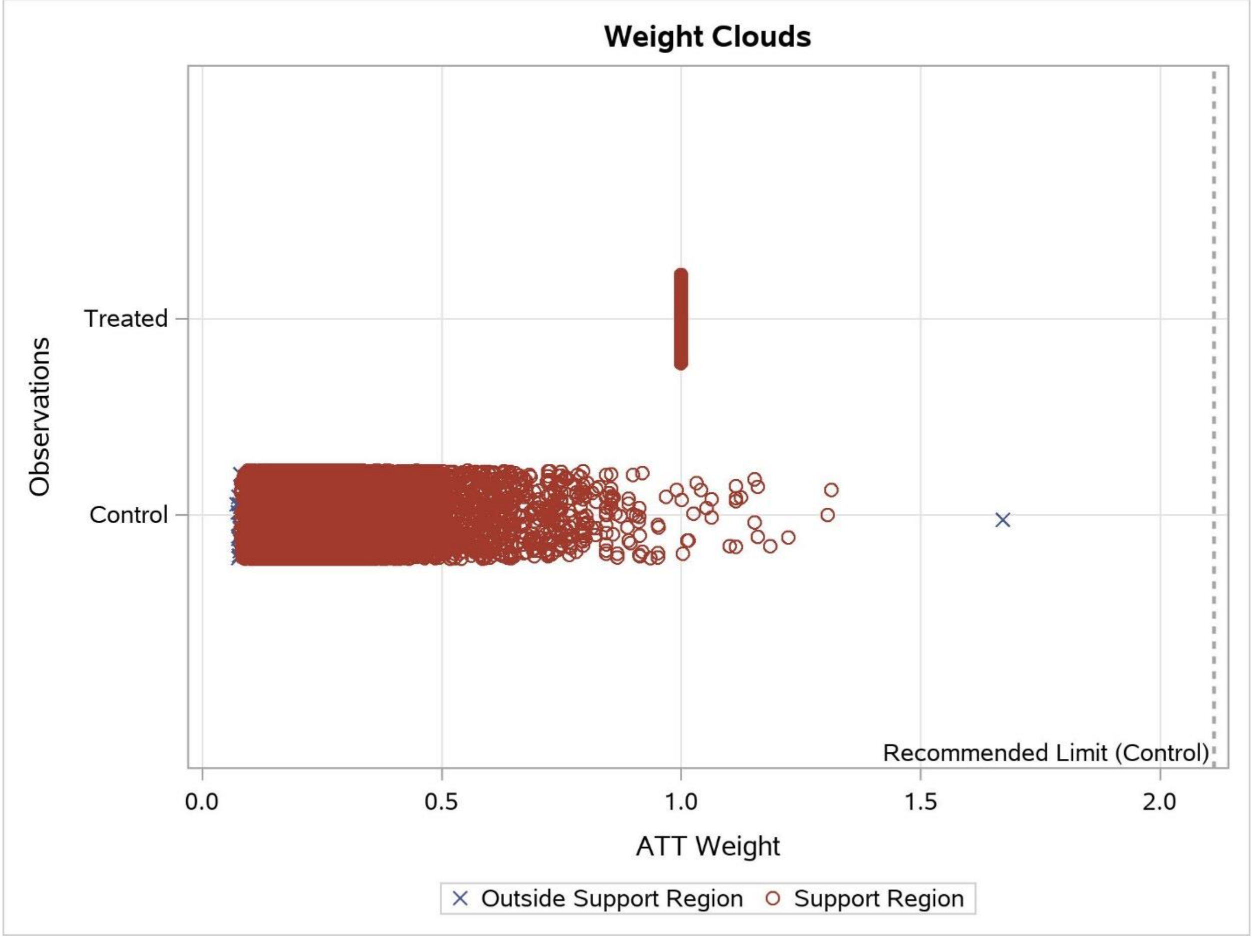

eFigure 4- The hazard function plot for the contraception failure in each study cohort

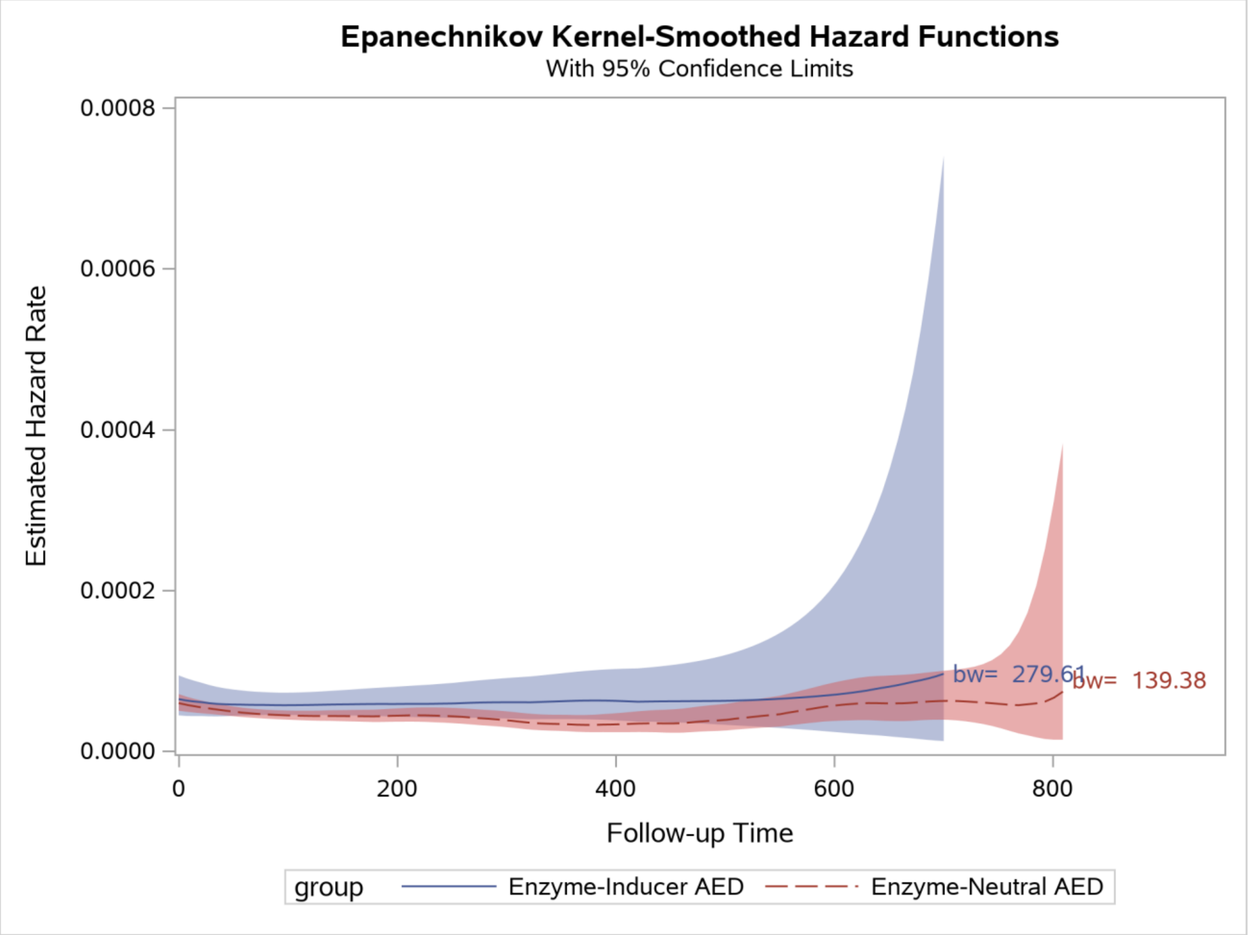

Supplement: Supplementary file 1 [file ede-32-268-s001.pdf]
