# Supplementary material for: A Pharmacoepidemiologic Approach to Evaluate Real-world Effectiveness of Hormonal Contraceptives in the Presence of Drug–drug Interactions
Source: Epidemiology. 2020 Nov 16;32(2):268–76. doi: 10.1097/EDE.0000000000001302 (PMC7850590; doi:10.1097/EDE.0000000000001302)
Supplement: Supplementary file 2 [file ede-32-268-s002.pdf]

Drug EI-AED + OC vs EI-AED alone

	TRUTH					OBSERVED	
	E1	E0	Sn	Sp		E1	E0
D+	35	1,150	1	0.95	D+	93	1,093
D-	3,465	10,350	1	0.95	D-	3,983	9,833
Total	3,500	11,500			Total	4,075	10,925
**OR**	**0.09**				**OR**	**0.21**	
**RR**	**0.10**				**RR**	**0.23**	
**RD**	**-0.09**				**RD**	**-0.08**	
**Risk**	**0.01**	**0.10**			**Risk**	**0.02**	**0.10**

Drug EN-AED + OC vs EN-AED alone

	TRUTH					OBSERVED	
	E1	E0	Sn	Sp		E1	E0
D+	210	6,000	1	0.95	D+	510	5,700
D-	20,790	54,000	1	0.95	D-	23,490	51,300
Total	21,000	60,000			Total	24,000	57,000
**OR**	**0.09**				**OR**	**0.20**	
**RR**	**0.10**				**RR**	**0.21**	
**RD**	**-0.09**				**RD**	**-0.08**	
**Risk**	**0.01**	**0.10**			**Risk**	**0.02**	**0.10**

Read me: Spread sheet for exploring the impact of sensitivity and specificity on comparative effectiveness studies of two active drugs. The user can enter values into the highlighted cells to control the sensitivty (Sn), specificity (Sp), total number of unexposed and exposed, the background risk and the relative risk (RR) for each drug compared to non-users.

Assumption:
1. The unit of analysis is one person-year in the tables. The numbers in the "OBSERVED" tables (E1 column) are approximately similar to the observed follow-up times in the study under 95% specificity.
2. Exposure time to EI-AED without OC is 3x than the concomitant use in the population.
2. Exposure time to EN-AED without OC is 3x than the concomitant use in the population.
3. The conception rate in the EI-AED or EN-AED alone groups are 10%.
4. The expected effectiveness of OC is 90% protection (RR=0.1).
5. In the "TRUTH" senario we assumed EI-ED or EN-AED have no effect on the expected effectiveness.

Drug EI-AED + OC vs Drug EN-AED + OC

	TRUTH			OBSERVED	
	Drug A	Drug B		Drug A	Drug B
D+	35	210	D+	93	510
D-	3,465	20,790	D-	3,983	23,490
Total	3,500	21,000	Total	4,075	24,000
**OR**	**1.00**		**OR**	**1.07**	
**RR**	**1.00**		**RR**	**1.07**	
**RD**	**0.00**		**RD**	**0.00**	
**Risk**	**0.01**	0.01	**Risk**	**0.02**	**0.02**
